# Birth weight and thoracoscopic approach for patients with esophageal atresia and tracheoesophageal fistula—a retrospective cohort study

**DOI:** 10.1007/s00464-024-11063-8

**Published:** 2024-07-17

**Authors:** Dominika Borselle, Sylwester Gerus, Monika Bukowska, Dariusz Patkowski

**Affiliations:** Department of Pediatric Surgery and Urology, Wroclaw Medical University, Borowska 213, 50-556, Wroclaw, Poland

**Keywords:** Thoracoscopy, Low birth weight, Esophageal atresia, Minimally invasive surgery, Newborn surgery

## Abstract

**Background:**

This study aimed to analyze the results, feasibility and safety of the thoracoscopic approach for patients with esophageal atresia with tracheoesophageal fistula (EA/TEF) depending on the patient’s birth weight.

**Methods:**

The study involved only type C and D EA/TEF. Among the analyzed parameters were the patients’ characteristics, surgical treatment and post-operative complications: early mortality, anastomosis leakage, anastomosis strictures, chylothorax, TEF recurrence, and the need for fundoplication or gastrostomy.

**Results:**

145 consecutive newborns underwent thoracoscopic EA with TEF repair. They were divided into three groups—A (*N* = 12 with a birth weight < 1500 g), B (*N* = 23 with a birth weight ≥ 1500 g but < 2000 g), and C—control group (*N* = 110 with a birth weight ≥ 2000 g). Primary one-stage anastomosis was performed in 11/12 (91.7%) patients—group A, 19/23 (82.6%)—group B and 110 (100%)—group C. Early mortality was 3/12 (25%)—group A, 2/23 (8.7%)—group B, and 2/110 (1.8%)—group C and was not directly related to the surgical repair. There were no significant differences in operative time and the following complications: anastomotic leakage, recurrent TEF, esophageal strictures, and chylothorax. There were no conversions to an open surgery. Fundoplication was required in 0%—group A, 4/21 (19.0%)—group B, and 2/108 (1.9%)—group C survivors. Gastrostomy was performed in 1/9 (11.1%)—group A, 3/21 (14.3%)—group B and 0%—group C.

**Conclusion:**

In an experienced surgeon’s hands, even in the smallest newborns, the thoracoscopic approach may be safe, feasible, and worthy of consideration. Birth weight seems to be not a direct contraindication to the thoracoscopic approach.

Some aspects of esophageal atresia with distal tracheoesophageal fistula (EA/TEF) repair remain contentious, with limited evidence for the best practice [1]. Based on the prevailing Gross classification of EA, type C—EA with distal TEF comprises approximately 84% of cases, while type D—EA with proximal and distal TEF constituting around 1% [2]. In the majority of these patients, primary anastomosis is feasible [3]. According to the European Reference Network for Rare Inherited Congenital Anomalies (ERNICA) consensus, the thoracoscopic approach for EA/TEF treatment has many advantages over the open approach but requires specialized expertise [1].

Minimally invasive surgery (MIS) has emerged as a promising approach for many procedures, including reconstructive ones, with particular significance for neonatal patients [1, 4]. The recent literature has revealed evidence that both thoracoscopic and open surgical strategies yield comparable outcomes in the treatment of EA/TEF [5–9]. Nevertheless, substantial concerns persist regarding the feasibility and safety of thoracoscopy in specific patient cohorts, notably those with birth weights below 2000 g and/or major associated malformations [8, 10]. The advancements in perinatal and anesthetic management have had a profound impact on better prognosis [11]. However, inherent challenges are primarily attributed to patient risk factors, including general instability, extremely limited operative space, fragile tissues, accompanying anomalies, and immature homeostasis mechanisms. Although higher mortality and morbidity rates have not been directly associated with performed open surgery, various strategies were described to minimize complications [12, 13]. Thoracoscopy in premature patients presents challenges including potential anesthetic intolerance to CO_2_ insufflation and technical complexities related to esophageal anastomosis [14]. Despite recent studies demonstrating favorable outcomes in thoracoscopic EA repair for low-birth-weight newborns, data remains limited due to the condition’s rarity, the complexity of minimally invasive surgery (MIS) and the extensive learning process essential for achieving proficiency and sufficient expertise in this technique [4, 14–19].

The main purpose of the study was to analyze the results, feasibility, and safety of the thoracoscopic approach for patients with EA/TEF, depending on the patient’s birth weight.

## Materials and methods

An entire population of the consecutive newborns underwent thoracoscopic EA/TEF repair at the Department of Pediatric Surgery and Urology in Wroclaw between 2005 and 2022. The study involved all newborns with type C and D EA/TEF who underwent surgical repair for EA/TEF at our department during the study period, encompassing patients born in other departments and subsequently transferred to our hospital. Long gap esophageal atresia (LGEA) cases, defined as type A and B, were excluded. Newborns who received their initial or definitive surgery at other departments were also excluded. The patients’ data were retrospectively reviewed from medical documentation. Patients were regularly followed up during scheduled visits at an outpatient clinic.

Newborns were divided into three groups according to birth weight: below 1500 g (Group A), equal to 1500 but below 2000 g (Group B), and equal or above to 2000 g (Group C—control group).

Throughout the study period all consecutive patients were operated on using a thoracoscopic approach. There were no conversions to open surgery in the entire series. From the first and successful thoracoscopic type C EA/TEF repair in Poland in 2005, which was performed in our department, the indication for thoracoscopic EA/TEF repair has been almost any case considered for open repair. The operative treatment was in majority of cases performed on the second—third day of life following appropriate preoperative management. The surgical procedure commenced with a preoperative rigid bronchoscopy to identify a potential proximal TEF, assess for possible malformations of trachea and larynx and predict the gap between esophageal pouches by position of the distal TEF. Throughout the thoracoscopic procedure bilateral lung ventilation was managed in all cases. The pneumothorax pressure was around 5–6 mmHg, depending on birth weight, and reduced to even 4 mmHg later, if a mediastinum exposure was sufficient. The procedure was performed using two 3 mm instrument ports and a 5 mm optic port with a 30-degree oblique telescope placed around the outer edge of the scapula. In cases of fistula closure with a clip, there were one 3 mm and two 5 mm trocars [20]. The surgical treatment involved distal (and in type D also proximal) TEF closure and anastomosis between esophageal pouches. Esophageal anastomosis was performed over a 6–8 Fr nasogastric (NG) tube with single, sliding-knot absorbable 4–0 or 5–0 sutures. In almost all cases, it was a one-staged thoracoscopic management. A few newborns with type C required a two-staged EA/TEF repair because of intraoperative instability: TEF closure in the first stage, and esophageal anastomosis in the second stage.

At the beginning of the study period, TEF was commonly clipped, but later we transitioned to transfixing or ligating it with a suture. With growing experience and evolution of the technique we stopped to use electrosurgery for tissue dissection, suction, and irrigation during the procedure. Initially, all patients had a chest drain inserted, but nowadays, only occasionally, e.g., uncertain anastomosis or suspicion of air lung leakage. In all cases, the azygos vein was preserved with the anastomosis above or under the vein depending on distal fistula position. After the procedure patients routinely stayed in the intensive care unit on ventilator for couple of days.

The analyzed post-operative complications encompassed anastomotic leakage, anastomotic strictures, chylothorax, TEF recurrence, need for fundoplication or gastrostomy, as well as early and late mortality. All patients underwent an X-ray contrast study, usually on the 5th–6th post-operative day. If there had been no anastomotic leakage, the oral feeding would have been started, and if successful, the NG tube would have been removed. Any leakage was treated conservatively, if necessary, with pleural drainage. Anastomosis strictures were divided into these ones requiring single or multiple dilatations. All cases of anastomotic strictures were treated through endoscopic dilatations only in symptomatic cases. Early and late mortality was defined as mortality before and after discharge, respectively. Some patients required a Nissen fundoplication procedure due to resistant to conservative treatment symptoms of gastroesophageal reflux during the long-term follow-up. There were also patients who required gastrostomy placement due to oral feeding difficulties.

Verification of the normality of quantitative variables was performed using the Shapiro–Wilk test. Quantitative variables were reported as mean values ± standard deviation or, if not normally distributed, as median, inter-quartile ranges and minimum and maximum values, while qualitative variables were reported as numbers and percentages. Quantitative variables were compared using the Kruskal–Wallis test and the Dunn’s test, while categorical variables were compared using the chi-square test or Fisher’s exact test. For contingent tables, odds ratios and their 95% confidence intervals were estimated. The operative time in relation to the time since the first surgery was analyzed using the segmented regression model. Threshold value of birth weight to estimate a probability of early mortality was determined using ROC curve—Youden’s index, AUC (area under the curve) and cut-off point were determined. The *P*-values < 0.05 were considered statistically significant.

The study was conducted according to STROBE guidelines.

This study was approved by the Ethics Committee of the Medical University in Wroclaw with the Approval Code of 169/2022 and Approval Date 24.02.2022.

## Results

The entire population of 145 consecutive patients, who were operated on between 2005 and 2022, were included. The study cohort comprises 143 cases with type EA/TEF and 2 cases with type D EA/TEF. The surgeries were performed by the two operators experienced in newborn thoracoscopy. There was no open repair for primary cases in the study period (Table 1).

**Table 1:** Characteristics of the study population (gender, gestational age, birth weight and accompanied anomalies)

Variables	Birth weight (g)			Result of the test
	< 1500	1500–2000	≥ 2000	
	Group A (*N* = 12)	Group B (*N* = 23)	Group C (control) (*N* = 110)	
Gender				*χ*^2^ = 0.88 *df* = 2 *p* = 0.646^b^
Female, *N* (%)	7 (58.3%)	10 (43.5%)	49 (44.1%)	
Male, *N* (%)	5 (41.7%)	13 (56.5%)	61 (55.4%)	
Birth weight (g)				*F* = 108.7 *df*_1_ = 2 *df*_2_ = 142 ***p* < 0.001**^a^
M ± SD	1.22 ± 0.2	1.72 ± 0.15	2.77 ± 0.49	
Me [Q1; Q3]	1.22 [1.05; 1.42]	1.72 [1.55; 1.85]	2.7 [2.35; 3.1]	
Min–Max	0.9–1.46	1.5–1.95	2.0–3.9	
Gestational age (completed weeks)				*F* = 77.7 *df*_1_ = 2 *df*_2_ = 142 ***p* < 0.001**^a^
M ± SD	31.9 ± 1.6	33.5 ± 2.3	37.7 ± 2.2	
Me [Q1; Q3]	31 [31; 33]	34 [32; 35]	38 [37; 39]	
Min–Max	30–35	27–37	31–42	
Isolated/associated EA/TEF				*χ*^2^ = 6.84 *df* = 2 ***p* = 0.033**^b^
Isolated	6 (50.0%)	8 (34.8%)	70 (63.6%)	
Associated	6 (50.0%)	15 (65.2%)	40 (36.4%)	
Associated malformations				
Major cardiac	3 (25.0%)	6 (26.1%)	20 (18.2%)	*p* = 0.623^c^
Major renal	1 (8.3%)	4 (17.4%)	7 (6.4%)	*p* = 0.218^c^
Trisomy 18	2 (16.7%)	1 (4.4%)	1 (0.9%)	***p* = 0.006**^c^
Other genetic	1 (8.3%)	4 (17.4%)	10 (9.1%)	*p* = 0.479^c^

*p*-values < 0.05 were considered statistically significant

*M* mean, *SD* standard deviation, *Me* median, *Q1* lower quartile, *Q3* upper quartile

Test: ^a^ANOVA, ^b^Chi squared, ^c^Fisher’s exact

A significant positive correlation between birth weight and gestational age was observed. An increase of 1 week in gestational age was accompanied by average of 187 g increase in birth weight. Associated malformations were significantly more frequent in patients with birth weights lower than 2000 g.

All repairs were performed using thoracoscopic technique. There were no conversions to an open surgery. In almost all patients it was right-side thoracoscopic approach, even in cases with right aortic arch. We identified 10/145 (6.9%) patients with right aortic arch. One patient with right lung agenesia required left-side thoracoscopic repair. In all patients the native esophagus was preserved without any requirement for esophageal replacement. The azygos vein was preserved in all cases. In the first 33 patients, electrosurgery was used for tissue dissection, and in 1 patient due to minimal local bleeding. In the others, there was no need for any electrosurgery, suction, or irrigation, and only blunt tissue dissection, seldom aid with scissors was performed to mobilize the esophageal pouches. The intraoperative blood loss was hardly any, so there was no case that required blood transfusion due to surgery.

The majority of cases were treated in one stage. Five patients required a two-staged procedure due to respiratory instability: the TEF closure at the first stage, and the esophageal anastomosis during the second stage several days later (from 4 to 39 days, median 7 days). Gastrostomy tube placement was performed in three patients later due to feeding problems and in one patient with low birth weight and concomitant duodenal atresia during the initial surgery that involved only TEF ligation (esophageal anastomosis was scheduled later) and duodenal anastomosis for early initiation of enteral feeding. Multi-staged treatment was significantly more often necessary in newborns with birth weights < 2000 g. A full anastomosis was accomplished in all cases except for one patient with Edwards syndrome due to early mortality. The patient with a birth weight of 900 g had an earlier iatrogenic perforation of the upper pouch that was closed along with TEF ligation using the thoracoscopic approach. Subsequently, the patient died of circulatory-respiratory failure on the fourth day after surgery (Table 2).

**Table 2:** Illustrated day of life at first surgery, number of surgical stages, postoperative complications, operative times (operative time involved only thoracoscopic esophageal anastomosis, without the time needed for preparation and bronchoscopy or first stage with fistula ligation) and operative team details

Variables	Birth weight (g)			Results of the test
	Group A < 1500 *N* = 12	Group B 1500–2000 *N* = 23	Group C > 2000 *N* = 110	
	*N* (%)	*N* (%)	*N* (%)	
Day of life at first surgery				*H* = 0.56 *df*_1_ = 2 *df*_2_ = 144 *p* = 0.430^a^
*M* ± *SD*	3.0 ± 1.5	2.5 ± 1.3	2.8 ± 1.8	
*Me* [*Q*1; *Q*3]	3 [2; 3]	2 [2; 3]	2 [2; 3]	
*Min*–*Max*	1—11	1—6	1—9	
Number of surgery stages				*χ*^2^ = 18.2 *df* = 2 ***p* < 0.001**^b^
1	11 (91.7%)	19 (82.6%)	110 (100.0%)	
2	1 (8.3%)	4 (17.4%)	0 (0.0%)	
Postoperative complications				
Early mortality	3 (25.0%)	2 (8.7%)	2 (1.8%)	***p* = 0.001**^c^
Late mortality	0 (0.0%)	2 (8.7%)	3 (2.7%)	*p* = 0.286^c^
Anastomosis leakage	2 (18.2%)	1 (4.4%)	7 (6.4%)	*p* = 0.294^c^
	*N* = 9^*^	*N* = 21^*^	*N* = 108^*^	
One-time esophageal stricture	1 (11.1%)	1 (4.8%)	8 (7.4%)	*p* = 0.684^c^
Recurrent esophageal strictures	2 (22.2%)	8 (38.1%)	25 (23.1%)	*p* = 0.359^c^
Chylothorax	1 (11.1%)	2 (9.5%)	2 (1.9%)	*p* = 0.087^c^
TEF recurrence	0 (0.0%)	0 (0.0%)	1 (0.9%)	*p* = 1.000^c^
Fundoplication	0 (0.0%)	4 (19.0%)	2 (1.9%)	***p* = 0.009**^c^
Gastrostomy	1 (11.1%)	3 (14.3%)	0 (0.0%)	***p* = 0.003**^c^
Tracheostomy	1 (11.1%)	1 (4.8%)	1 (0.9%)	*p* = 0.066^c^
Operative time (min.)	*N* = 11^*^	*N* = 22^*^	*N* = 95^*^	*H* = 1.15 *df*_1_ = 2 *df*_2_ = 127 *p* = 0.563^a^
*M* ± *SD*	90 ± 28	99 ± 44	96 ± 30	
*Me* [*Q*1; *Q*3]	79 [70; 106]	81 [71; 120]	90 [76; 110]	
*Min*–*Max*	56–150	52–245	46–245	
Operative team	*N* = 12	*N* = 23	*N* = 110	*χ*^2^ = 6.50 *df* = 4 *p* = 0.165^b^
Operator A + Assist	11 (91.7%)	19 (82.6%)	71 (64.5%)	
Operator B + Assist	0 (0.0%)	1 (4.4%)	17 (15.5%)	
Operator A and Operator B	1 (8.3%)	3 (13.0%)	22 (20.0%)	

*p*-values < 0.05 were considered statistically significant

^*^Overall number of complications was analyzed in the population after excluding cases of early deaths

Test: ^a^Kruskal–Wallis, ^b^Chi squared, ^c^Fisher’s exact

The study revealed a statistically significant association between birth weight and early mortality, as well as between birth weight and the need for fundoplication and gastrostomy.

We had three cases at the initial series that required temporary tracheostomy placement due to severe tracheomalacia.

There were two major complications during the surgery. In one case during the distal fistula dissection, the left bronchus was opened. It was immediately noticed and closed with interrupted sutures. In another case during the procedure, the aorta was incorrectly ligated instead of the fistula. The suture was removed shortly when it was diagnosed without adverse consequences.

Age at early death ranged from 0 to 28 days (7 cases, median 4 days). Early mortality was mainly connected to patient factors—associated malformations and perioperative morbidity. There was one case of death caused by bilateral pneumothorax after reintubation on the second postoperative day. It was assumed that the fistula site closure was perforated by the intubation tube tip (Table 3).

**Table 3:** Number of early mortality cases with causes in the three groups of patients with odds ratios (OR)

Birth weight (g)	Early mortality		Result of the test	Cause of early mortality	OR (95% CI)	RR
	Yes *N* = 7	No *N* = 138				
< 1500	3 (42.9%)	9 (6.5%)	*χ*^2^ = 13.5 *df* = 2 ***p* = 0.001**	1. Trisomy 18 (2007) 2. Trisomy 18, upper pouch perforation before surgery (2009) 3. Multiorgan dysfunction (2020)	**18.0 (2.65–122)**	**13.74**
1500–2000	2 (28.6%)	21 (15.2%)		1. Major cardiac defect, systemic infection (2005) 2. Trisomy 18 (2007)	**5.15 (0.69–38.6)**	**4.78**
> 2000	2 (28.6%)	108 (78.3%)		1. Multiple defects, including major cardiac (2011) 2. Bilateral pneumothorax after reintubation (2013)	1.00 (ref.)	1.00 (ref.)

*p*-values < 0.05 were considered statistically significant

A ROC curve, which was prepared to estimate the probability of early mortality based on birth weight, revealed a cut-off point of 1.7 kg (AUC = 0.791, Youden’s index = 0.58).

The early mortality rate and anastomotic leakages significantly decreased between the first and the second half of the analyzed term (Table 4).

**Table 4:** Early mortality cases, number of anastomotic leakages, and number of esophageal strictures in relation to the number of patients operated on for EA/TEF during the two periods of time

Variables	Period of time		Fisher’s exact test	OR (95% CI)
	2005–2013 *N* = 56	2014–2022 *N* = 89		
Number of early deaths	6 (10.7%)	1 (1.1%)	***p* = 0.014**	**10.56 (1.236–90.232)**
Number of anastomotic leakages	8 (14.3%)	2 (2.2%)	***p* = 0.008**	**7.25 (1.480–35.520)**
	*N* = 50^*^	*N* = 88^*^		
Overall number of esophageal strictures	12 (24.0%)	33 (37.5%)	*p* = 0.131	0.526 (0.241–1.148)

*p*-values < 0.05 were considered statistically significant

^*^Overall number of esophageal strictures was analyzed in the population after excluding cases of early deaths

The percentage of other complications was similar between groups. All cases of anastomotic leakage were treated conservatively with adequate chest drainage. There was no requirement for reoperation. All esophageal strictures were treated with endoscopic dilatation. Endoscopic dilatation was sufficient in all esophageal strictures.

During the follow-up, six patients developed gastroesophageal reflux with severe symptoms resistant to conservative treatment, requiring fundoplication. The indication for gastrostomy placement and anti-reflux surgery, was statistically significant in newborns with birth weights below 2000 g. Notably, only one patient with a birth weight below 2000 g necessitated both fundoplication and gastrostomy placement due to feeding difficulties during the follow-up period.

**Fig. 1:**
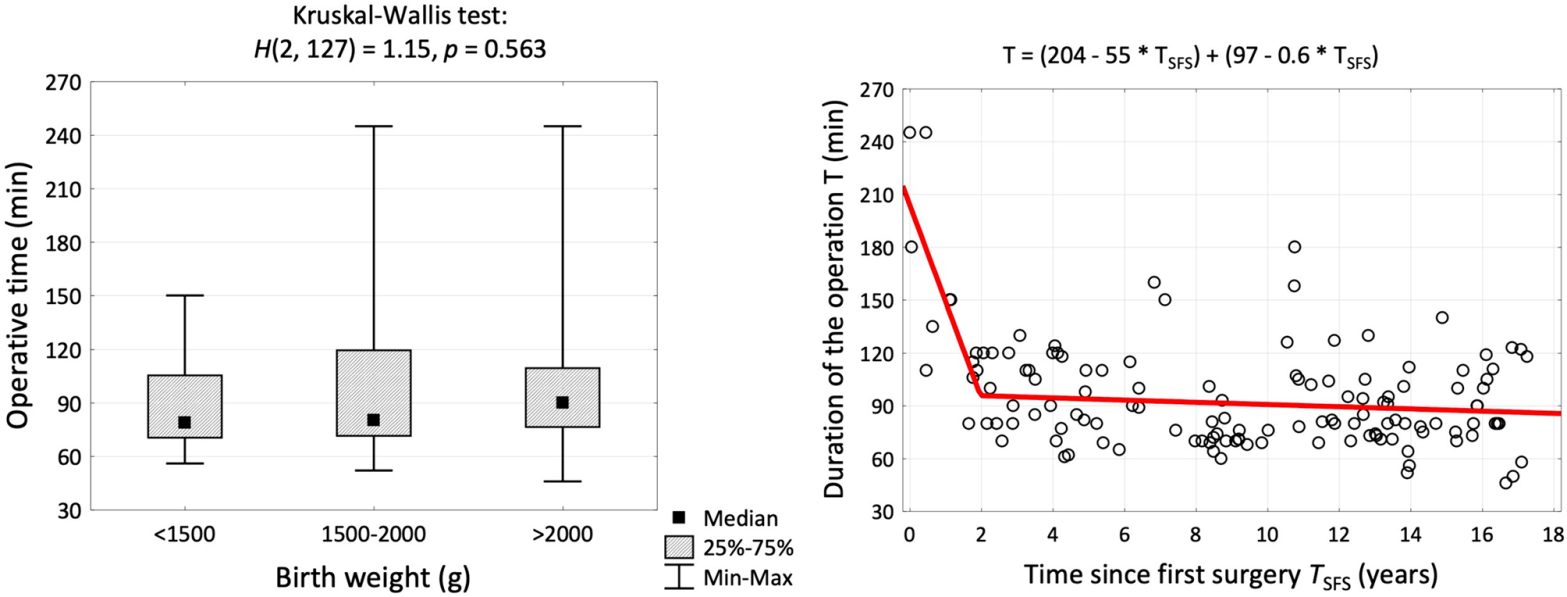
The association between birth weight and operative time and Kruskal–Wallis Test. Scatterplot of operative time (min) versus time since the first surgery (years) and segmented regression model

Operative time extended from 46 to 245 min with a median of 89 min. There was no significant association between operative time and the patient’s birth weight. Operative time was associated with the number of performed EA/TEF repairs over time. Operative time plummeted by about 55 min per year over the first 2 years, then the rate of decline significantly flattened down and stabilized at the median of 82 min (Fig. 1).

## Discussion

The first thoracoscopic EA with TEF repair was performed by S. Rothenberg in 2000 and since then it has been gradually becoming more widely utilized [21]. However, an adoption of the technique is still controversial in some groups of patients [8, 10]. Among contraindications to the thoracoscopic approach for EA/TEF, the literature pointed out severe hemodynamic instability as an absolute contraindication, while relative contraindications comprised significant cardiac defects, prematurity, low birth weight and LGEA cases [8, 22]. Newborns with birth weights below 2000 g constitute approximately a quarter of the entire EA/TEF population. Birth weight is considered one of the primary determinants in prognostic classifications, stratifying the survival rate, therefore the optimal surgical management, approach and timing for esophageal anastomosis remain subjects of debate [3, 23].

Newborns with low birth weight are more commonly observed to have associated major malformations, including life-threating chromosomal and heart defects [24, 25]. These anomalies can significantly impact a patient’s hemodynamic stability, increase the likelihood of complications, and influence decisions on surgical and anesthetic strategy. Some studies have proposed that the presence of major cardiac and chromosomal anomalies may pose a greater risk of mortality than just low birth weight [11, 26, 27]. In our study, major cardiac defect was responsible for almost a third of early mortality cases. Furthermore, low-birthweight, premature newborns are susceptible to multi-organ morbidity, that may deteriorate the survival rate. Newborns with EA may also present with concomitant airway malformations, including tracheomalacia or laryngeal cleft, which may contribute to respiratory insufficiency requiring tracheostomy, especially in low-birth-weight neonates.

In a recent multi-center study, early mortality rate was found to exceed 10% and was correlated with lower birth weight and concomitant anomalies [28]. Similarly, our observations indicated a substantial association between low birth weight and early mortality, which was not directly related to the surgical repair. Supporting this, Zani et al. revealed that mortality among extremely low birth weight neonates stemmed from complications unrelated to the surgical repair [12]. Furthermore, we noted a considerable decrease in the early mortality rate over time. The overall enhancements in both survival rates and successful esophageal repairs may reflect advancements in perinatal care and surgical proficiency [25, 29, 30]. Our data, along with existing literature suggests that birth weight above 1700 g and gestational age above 33.5 weeks at the time of surgery may be associated with a reduced risk of early death [31].

The advantages and disadvantages of MIS approach for EA/TEF are the next aspects to discuss. All consecutive patients in our study were treated solely by the thoracoscopic approach with no conversions to open surgery and primary anastomosis was feasible in almost every patient. Thoracoscopy, as a technique of minimal access, diminishes injury to the chest wall, that is particularly advantageous for the smallest neonates, and results in less thoracic musculoskeletal deformities in the long-term follow-up [4, 10, 21, 32–39]. The precise visualization capability of thoracoscopy enhances its utility in restricted, hard-to-access operative spaces, therefore an esophageal mobilization and an anastomosis high in the chest may be simpler compared to open approach [10, 21, 32]. Due to magnified vision and minimal tissue manipulation, the likelihood of postoperative complications such as tracheomalacia or injury to the tracheal membranous wall and surrounding tissues may be decreased [21]. Visualizing the thoracic inlet facilitates the safe management of high or upper pouch fistulas in type D EA/TEF, that may eliminate the necessity for a neck incision [40, 41].

Among indications for conversion the literature pointed out the intraoperative adverse effects or instability [42]. Newborns weighing < 2000 g more frequently demonstrated respiratory instability. The preterm, unstable neonates in our series were treated with two-staged thoracoscopic EA/TEF repair, aimed to stabilize the patient’s condition by TEF closure during the first stage, followed by esophageal anastomosis in the second stage [20]. It is worth to notice that the majority of patients tolerated the thoracoscopic approach very well. The literature reported that the strategy of staged open repair, involving gastrostomy placement and delayed anastomosis in extremely premature newborns was associated with a significantly reduced incidence of anastomotic complications [12, 30, 43]. However, a disadvantage of this management is the requirement for two thoracotomies, resulting in increased chest-wall injury and elevated adhesions formation, subsequently impeding the visualization of thoracic anatomy [36–38]. In contrast, thoracoscopic staged repair may allow to mitigate these disadvantages of open approach, and can be performed within shorter time intervals, thereby obviating the need for gastrostomy placement [20]. In this regard thoracoscopy may improve the management of type C EA/TEF in low birth weight, unstable newborns as it is supported by our data. In our department, thoracoscopy was performed even in the extremely low birth weight newborns and in all types of EA, including LGEA, with positive results [20].

Among the disadvantages of thoracoscopy the literature highlighted its technical complexity [10, 21]. It seems to be particularly attributed to the proficiency required in MIS suturing [21]. The complexity may be enhanced in newborns weighing less than 2 kg due to limited space, fragile tissues [44] and the potential anatomical variances in patients with associated malformations. The literature reported that the inability to complete an open primary esophageal anastomosis in newborns weighing less than 1500 g might be attributed to more subtle tissues [44]. It also pointed out that just the considerable tissue immaturity and vulnerability posed complexities of the anastomosis [30, 45]. However, in our series the complete anastomosis was feasible in almost all patients, primarily or just within a few days during the second stage, and the anastomotic complications were not correlated with birth weight.

Despite almost 25 years passing since the first thoracoscopic EA with TEF repair, the literature reported the conversion rate of up to 50% as shown by Etchill et al., in their analysis utilizing American NSQIP data [46]. Notably, data sourced from over 140 hospitals indicated a mean caseload of less than one case per hospital. These findings are consistent with statistics provided by the American Board of Surgery, indicating the median annual caseload of one EA/TEF case per surgeon in the United States, regardless of the surgical technique used [47]. In the meta-analysis of Drevin et al., including 1047 EA patients, thoracoscopy was applied in 33% with conversion rate of 10% [48]. At our center, the average case-volume has progressively increased from 8.5 cases per year to 14.5 cases per year over the last five years, specifically for the primary type C cases, which were analyzed. Our results indicate a potential to completely avoid the need for conversion, which differs from the findings in the existing literature. So far, we have not had a conversion, which we refer to our experience with the technique resulting from the number of operated cases and reducing the operating team to two surgeons proficient in newborn thoracoscopy, of whom at least one is always present during the procedure. Additionally, we have successfully performed numerous staged thoracoscopic repairs for LGEA patients not included in this study [49]. Our center has also managed a considerable number of secondary complicated cases from other centers, often presenting with complexities and associated malformations. A crucial aspect to note is the extensive experience of the Operator A (DP), who has performed in summary over 400 thoracoscopic EA repairs across various healthcare institutions in Poland and globally, that underscores the department’s proficiency in performing these surgical interventions. According to the literature, complication rate might have been lowered by reducing the number of operators in difficult cases, which reflects the crucial role of surgical expertise and the strategic decision-making [50].

Furthermore, our study reported a similar rate of post-operative complications across the birth-weight subgroups, except for the requirement for anti-reflux surgery and gastrostomy placement. Feeding difficulties and dysphagia in the long-term follow-up mainly affected children with a history of prematurity, low birth weight and comorbidities. However, our results demonstrated significantly lower frequency of gastrostomy and anti-reflux operations compared to those presented in the literature [51]. In our material, only three patients were diagnosed with severe tracheomalacia requiring tracheostomy within the initial series. Interestingly, despite the cohort’s size, aortopexy was performed in one patient. These results we can refer not only to the experience, but to the technique of precise blunt dissection, without any electrocoagulation. In our opinion, this method serves to prevent harm to surrounding tissues, thereby minimizing the subsequent fibrosis. Preserving the smallest vessels and innervation is hypothesized to potentially influence later esophageal motility, however this is only our assumption, which requires further observations and research [20]. Son et al. also outlined that most post-operative outcomes following thoracoscopic EA repair, displayed no difference among study cohorts, except for a considerably higher prevalence of gastroesophageal refluxes requiring fundoplication in the population of birth weight below 2000 g [19]. While an association between birth weight and anastomotic complications in our study was not observed within the entire population, anastomotic leakage was significantly more frequent during the first half of the study period that may be connected to the growing experience.

According to the literature, the early post-operative complications and operative time may reflect the effect of increasing experience of the surgeon [15–19, 52]. In this study the experience was therefore considered as corresponding to the number of performed EA/TEF repairs, patient outcomes and operative time. However, not only EA/TEF repairs, but also other thoracoscopic interventions have continually building the surgeon’s expertise. Operative time has been considered in the literature as a disadvantage of MIS for EA/TEF repair [32]. In our study, operative times were longer for the first procedures in the entire series and progressively decreased, notably within the first two years, possibly reflecting a learning curve pattern [52]. The operative procedures were carried out with a median operative time of 89 min, frequently achieving durations below or around 60 min, with no observed correlation with the patient birth weights. In contrast, the operative times for thoracoscopic procedures were notably longer in studies by Etchill (217 min) and Drevin (149–244 min), that might be associated with learning curve, the caseload per surgeon and, consequently affect the conversion rate [46, 48]. Therefore, efforts should be made to diminish the impact of the learning curve [16, 17, 19, 53].

Arguably, management of the full spectrum of EA patients with MIS, including the smallest newborns, LGEA, and complex cases, may require a centralization in surgical departments for better outcomes [54]. Similarly, the recent studies reported positive results with thoracoscopic approach for EA/TEF, regardless of birth weight and in the majority of EA types, including LGEA [3, 14, 20, 21, 50, 52]. Moreover, the literature found no confirmation that thoracoscopic approach compromised outcomes in patients with congenital heart disease [14, 55]. Twenty percent of patients in our series presented major cardiac malformations, and thoracoscopy was proceeded without complications in all of them.

Although we have conducted the research on surgical aspects of the management of EA with TEF and low birth weight, the comprehensive evaluation should also include anesthetic treatment. Hypercapnia, acidosis and cerebral oxygenation among patients undergoing thoracoscopic procedures have been areas of concern, however existing literature has not proved negative fluctuations in these parameters during the operation [10, 56, 57]. Other authors highlighted the complexities of general anesthesia in EA/TEF newborns with major cardiac anomalies and low birth weights [20, 58, 59]. Nevertheless, the recent study found no significant variances in intraoperative parameters or severe events occurrence between patients with normal and low birth weights [19]. The strong side of this study is a large, homogenous population of consecutive patients treated only by thoracoscopic approach and by one of the two experienced surgeons. Reliable and complete evaluation of safety and feasibility of thoracoscopy may be advantageous with regard to the development of new MIS technologies to fully exploit their potential in the most complex cases.

Thoracoscopy can be a safe technique in experienced surgeons’ hands, even in the smallest newborns and patients with congenital heart disease. Based on our experience birth weight seems to be not a direct contraindication to the thoracoscopic approach [10, 14, 19].
